# Incidence of gestational diabetes mellitus in the United Arab Emirates; comparison of six diagnostic criteria: The Mutaba’ah Study

**DOI:** 10.3389/fendo.2022.1069477

**Published:** 2022-12-12

**Authors:** Maryam M. Bashir, Luai A. Ahmed, Iffat Elbarazi, Tom Loney, Rami H. Al-Rifai, Juma M. Alkaabi, Fatma Al-Maskari

**Affiliations:** ^1^ Institute of Public Health, College of Medicine and Health Sciences, United Arab Emirates University, Al Ain, United Arab Emirates; ^2^ Zayed Centre for Health Sciences, United Arab Emirates University, Al Ain, United Arab Emirates; ^3^ College of Medicine, Mohammed Bin Rashid University of Medicine and Health Sciences, Dubai, United Arab Emirates; ^4^ Department of Internal Medicine, College of Medicine and Health Sciences, United Arab Emirates University, Al Ain, United Arab Emirates

**Keywords:** gestational diabetes mellitus, incidence, IADPSG, diabetes, risk factors, diagnostic criteria, United Arab Emirates

## Abstract

**Background:**

For more than half a century, there has been much research and controversies on how to accurately screen for and diagnose gestational diabetes mellitus (GDM). There is a paucity of updated research among the Emirati population in the United Arab Emirates (UAE). The lack of a uniform GDM diagnostic criteria results in the inability to accurately combine or compare the disease burden worldwide and locally. This study aimed to compare the incidence of GDM in the Emirati population using six diagnostic criteria for GDM.

**Methods:**

The Mutaba’ah study is the largest multi-center mother and child cohort study in the UAE with an 18-year follow-up. We included singleton pregnancies from the Mutaba’ah cohort screened with the oral glucose tolerance test (OGTT) at 24–32 weeks from May 2017 to March 2021. We excluded patients with known diabetes and with newly diagnosed diabetes. GDM cumulative incidence was determined using the six specified criteria. GDM risk factors were compared using chi-square and t-tests. Agreements among the six criteria were assessed using kappa statistics.

**Results:**

A total of 2,546 women were included with a mean age of 30.5 ± 6.0 years. Mean gravidity was 3.5 ± 2.1, and mean body mass index (BMI) at booking was 27.7 ± 5.6 kg/m^2^. GDM incidence as diagnosed by any of the six criteria collectively was 27.1%. It ranged from 8.4% according to the EASD 1996 criteria to 21.5% according to the NICE 2015 criteria. The two most inclusive criteria were the NICE 2015 and the IADPSG criteria with GDM incidence rates of 21.5% (95% CI: 19.9, 23.1) and 21.3% (95% CI: 19.8, 23.0), respectively. Agreement between the two criteria was moderate (k = 0.66; p < 0.001). The least inclusive was the EASD 1996 criteria [8.4% (95% CI: 7.3, 9.6)]. The locally recommended IADPSG/WHO 2013 criteria had weak to moderate agreement with the other criteria, with Cohen’s kappa coefficient ranging from (k = 0.51; p < 0.001) to (k = 0.71; p < 0.001). Most of the GDM risk factors assessed were significantly higher among those with GDM (p < 0.005) identified by all criteria.

**Conclusions:**

The findings indicate discrepancies among the diagnostic criteria in identifying GDM cases. This emphasizes the need to unify GDM diagnostic criteria in this population to provide accurate and reliable incidence estimates for healthcare planning, especially because the agreement with the recommended criteria was not optimal.

## Introduction

For over half a century, there have been many controversies on the standard way to screen for and diagnose gestational diabetes mellitus (GDM) among pregnant women, yet there is still no single globally acceptable guideline for this purpose. Lack of evidence, availability of resources, convenience, different expert opinions, differences between populations’ risks, and many other reasons have contributed to this challenge (1, 2). The prevalence of GDM in the United Arab Emirates (UAE) ranges from 7.9% to 24.9% (3) and, in some cases, up to 37.7% (4). These variations are due to different diagnostic criteria, the timing of screening, screening methods, and sub-populations, among other factors (5).

Over the years, globally, different diagnostic criteria and recommendations for screening and diagnosing GDM have been published. Most widely used criteria include the International Association of Diabetes and Pregnancy Study Groups (IADPSG 2010) (6), World Health Organization (WHO 2013) (7), WHO (1999) (8), the American Diabetes Association (ADA 2018) (9), the Australasian Diabetes in Pregnancy Society (ADIPS 1998) (10), the National Institute for Health and Clinical Excellence (NICE 2015) (11), the Canadian Diabetes Association (CDA 2013) (12), Carpenter and Coustan criteria (C&C 1982) (13), National Diabetes Data Group (NDDG 1979) (14), European Association for the Study of Diabetes (EASD 1996) (15), New Zealand Society for the Study of Diabetes criteria (NZSSD) (16), and the International Federation of Gynecology and Obstetrics (FIGO 2015) (17).

The IADPSG is currently one of the most acceptable and widely used criteria globally because it is based on the results of the Hyperglycemia and Adverse Pregnancy Outcomes (HAPO) study (18), which is a multi-center, multinational, blinded prospective cohort study and is potentially one of the most generalizable regarding this topic. Some guidelines have been updated according to the IADPSG recommendations [e.g., WHO 2013, FIGO 2015, ADIPS 2017, ADA 2018 (alternate), and CDA 2013 (alternate)], although many others have not (2). Lack of a uniform standardized global guideline results in the inability to accurately combine or compare the disease burden worldwide or even at a local level and develop a simple, standardized GDM management protocol that could be applied globally (1).).

Different diagnostic criteria have been found to classify GDM differently (19–25). In the Gulf region, a recent study in Oman showed that 48.5% of patients with GDM were identified by the IADPSG (WHO 2013) criteria and only 26.4% by the former WHO 1999 criteria (26). Meanwhile, a study in Qatar showed that 21.5% of patients with GDM were identified by the WHO 2013 criteria (IADPSG) and 20.1% by the NICE criteria, with a kappa coefficient of 0.67 showing moderate agreement between the two criteria (27). The IADPSG criteria generally diagnose more patients with GDM than the other criteria (28). A study conducted in the UAE in 2005 showed that the ADIPS criteria were the most inclusive in diagnosing GDM at the time (3), while 10 years later, in a similar population, the IADPSG was found to be the most inclusive, with GDM prevalence rate ranging from 9.2% to 45.3% using different criteria (29). These studies were conducted among multi-ethnic women.

The effect of ethnicity on GDM has been well researched at the local and global levels showing varying GDM risks across different ethnicities (30, 31). The Emirati population, which is the local population of the UAE, has been described to have one of the highest prevalence of cardiovascular risk factors reported in the country and worldwide (32). However, very few studies have been conducted in this population to describe the effect of different diagnostic criteria on GDM incidence. This study aims to compare the incidence of GDM using the IADPSG, WHO 1999, NICE 2015, ADIPS 1998, EASD 1996, and NZSSD 2004 criteria among the Emirati population in the UAE. It also assesses the GDM risk factor distribution according to each GDM criterion and compares agreement between the different criteria used.

## Materials and methods

### Study design and setting

The Mutaba’ah study is the largest ongoing prospective mother and child cohort study in the UAE, recruiting women from the Emirati population during pregnancy and following them up during antenatal, birth, and postnatal periods and their children until the age of 18 years. It is being conducted in the city of Al Ain, Abu Dhabi Emirate, UAE, which has the highest proportion (30.8%) of Emirati Nationals in the country, of which women constitute 49%. Recruitment of participants is from the two major tertiary public hospitals and the largest private maternity hospital in the city. Details of the Mutaba’ah Study, including the recruitment process, have been published elsewhere (33).

### Participants

This study analyzed data from the pregnant women (Mutaba’ah Mother and Child Cohort Study) recruited between May 2017 and March 2021. Those screened for GDM at 24 to 32 weeks (with at least one reading) were included in this analysis. Only singleton pregnancies were included. Those with pre-existing diabetes or fasting blood glucose (FBG) of ≥7 mmol/L and/or 2-h OGTT (oral glucose tolerance test) ≥ 11.1 mmol/L [i.e., newly diagnosed type 2 diabetes mellitus (DM) cases] were excluded.

### Sample size

A minimum sample of 707 participants will allow for the detection of a true proportion (37.7%) of GDM cases identified by IADPSG criteria (4), given an 80% power and a 1% alpha error and considering a non-response rate of 20%. Estimation was done using online OpenEpi version 3.01.

### Data collection and variables

Data were collected using a self-administered questionnaire and extraction from the medical records. The questionnaire was administered at 12–25 weeks of gestation to the participants by trained research assistants using a tablet containing the questionnaire link, which is directly uploaded to the study database upon completion. It assessed information including sociodemographic, past, and current pregnancy history, medical history, and other factors. The questionnaire was available in both English and Arabic versions. Medical records were used to obtain other information on the current pregnancy, including all anthropometric measurements, laboratory results (including OGTT results), and details of previous pregnancies. For this analysis, data utilized included participants age, gravidity, body weight, height, and body mass index (BMI) at booking; personal history of diagnosis with GDM; family history of type 2 DM; level of education; employment status; and OGTT results.

### GDM screening and diagnosis

GDM screening in the public and private recruiting hospitals was similar. The recommendation was for all pregnant women to undergo universal screening with 75-g 2-h OGTT at 24 to 28 weeks of pregnancy during routine antenatal care (ANC) visits. At the first visit (<24 weeks), all women undergo a fasting plasma glucose (FPG) test or a HbA1C test to detect patients with pre-existing diabetes who were then co-managed with the endocrinologists. For GDM diagnosis in this study, we used six different diagnostic criteria, which are part of the most widely used in the UAE (34), and they all endorse universal one-step screening with 75-g OGTT at 24 to 28 weeks of gestation as done in the recruiting hospitals. They include IADPSG [WHO 2013/FIGO 2015/ADIPS 2017/ADA 2018 (alternate)/CDA 2013 (alternate)], NICE 2015, WHO 1999 (NICE 2008), ADIPS 1998, EASD 1996, and NZSSD 2004. Standard definitions are described in **Table 1**.

**Table 1:** GDM screening and diagnostic criteria.

	Population to screen	Timing of screening	Type of screening test	No. of abnormal values	Fasting plasma glucose (mmol/L)	1-h OGTT (mmol/L)	2-h OGTT (mmol/L)	3-h OGTT (mmol/L)
**IADPSG/WHO 2013/FIGO 2015/ADIPS 2017/ADA 2018 (alternate)/CDA 2013 (alternate)**	**Universal** **screening**	**24–28 weeks**	**One step, 2 h, 75 g**	**≥1**	**5.1**	**10.0**	**8.5**	**-**
**WHO 1999/NICE 2008**	**Universal** **screening**	**24–28 weeks**	**One step, 2 h, 75 g**	**≥1**	7.0*	**-**	**7.8**	**-**
**NICE 2015/RCOG**	**Selective/Universal screening**	**24–28 weeks**	**One step, 2 h, 75 g**	**≥1**	**5.6**	**-**	**7.8**	**-**
**ADIPS 1998**	**Universal** **screening**	**24–28 weeks**	**One step, 2 h, 75 g**	**≥1**	**5.5**	**-**	**8.0**	**-**
**EASD 1996**	**Universal** **screening**	**24–28 weeks**	**One step, 2 h, 75 g**	**≥1**	**6.0**	**-**	**9.0**	**-**
**NZSSD 2004**	**Universal** **screening**	**24–28 weeks**	**One step, 2 h, 75 g**	**≥1**	**5.5**	**-**	**9.0**	**-**
**NZSSD 2014**	**Universal** **screening**	**24–28 weeks**	**Two steps, 2 h, 75 g**	**≥1**	**5.5**	**-**	**9.0**	**-**
**CDA 2013** **(preferred)**	**Universal** **screening**	**First visit**	**Two steps, 2 h, 75 g**	**≥2**	**5.3**	**10.6**	**9.0**	**-**
**ADA 2018**	**Universal** **screening**	**24–28 weeks**	**Two steps, 3 h, 100 g**	**≥2**	**5.3**	**10.0**	**8.6**	**7.8**
**C&C 1982/ACOG2013/ADA 2004**	**Selective** **screening**	**First visit**	**Two steps, 3 h, 100 g**	**≥2**	**5.3**	**10.0**	**8.6**	**7.8**
**NDDG 1979**	**Selective** **screening**	**First visit**	**Two steps, 3 h, 100 g**	**≥2**	**5.9**	**10.6**	**9.2**	**8.1**
**Modified NDDG**	**Selective** **screening**	**First visit**	**Two steps, 3 h, 100 g**	**≥2**	**5.3**	**10.1**	**8.7**	**7.8**

*Fasting plasma glucose threshold currently falls under the updated WHO criteria for existing diabetes mellitus. IADPSG, International Association of Diabetes and Pregnancy Study Groups; NICE, National Institute for Health and Clinical Excellence; WHO, World Health Organization; ADIPS, Australasian Diabetes in Pregnancy Society; EASD, European Association for the Study of Diabetes; NZSSD, New Zealand Society for the Study of Diabetes; RCOG, Royal College of Obstetricians & Gynaecologists; ACOG, American College of Obstetricians & Gynaecologists.

### Statistical analysis

Data analyses were conducted in Stata statistical software version 16.1 (StataCorp LLC, College Station, TX, USA). Continuous variables were summarized using means with standard deviations (SD), whereas the categorical variables were summarized using counts and proportions. GDM risk factors and other maternal characteristics were compared using chi-square test for categorical variables and *t*-test for continuous variables. An alpha level of significance was specified at 5%.

Cumulative incidence of GDM (by the six diagnostic criteria) from May 2017 to March 2021 was calculated as the number of pregnant women with GDM (as identified by a specific diagnostic criterion) divided by the total number of eligible pregnant women screened during the period multiplied by 100. Results were reported with their logit confidence intervals.

Agreements between the six diagnostic criteria were compared (in pairs) using kappa statistics. Cohen’s kappa coefficient (k) for each pair was reported. P-value was significant at <0.05.

Because of the missing values of some of the OGTT readings, we conducted sensitivity analysis to check for GDM cumulative incidences stratified by the number of non-missing OGTT readings used for diagnosis, i.e., those having at least one versus those having at least two OGTT readings.

## Results

A total of 2,586 pregnant women with singleton pregnancies were recruited and screened for GDM at 24 to 32 weeks of gestation during the study period. Thirty-nine patients with newly diagnosed diabetes and one patient with known diabetes were excluded. Hence, 2,546 patients were eligible to participate and included in the analyses.

### Participants’ characteristics

**Table 2** shows the maternal characteristics. The mean (± SD) age of the cohort was 30.5 ± 6.0 years, mean gravidity was 3.5 ± 2.1 pregnancies, and mean BMI at booking was 27.7 ± 5.6 kg/m^2^. Majority (94.7%) of the participants had at least a high school education, and 31.0% were employed. A fifth (20.6%) had previous GDM, and more than a quarter (29.6%) had a family history of type 2 DM. Their mean FPG was 4.6 ± 0.4 mmol/L, 1-h OGTT was 8.0 ± 1.9 mmol/L, and 2-h OGTT was 6.5 ± 1.6 mmol/L.

**Table 2:** Baseline characteristics of participants (N = 2,546).

Maternal Characteristics	Total Participants (N)^a^	Frequency [n (%)]	Mean ± SD
Age (years)	2,544		30.5 ± 6.0
Gravidity	2,546		3.5 ± 2.1
Educational status	2,345		
Primary and below High school Diploma Bachelors Postgraduate		125 (5.3) 975 (41.6) 244 (10.4) 912 (38.9) 89 (3.8)	
Employment status	2,348		
Student Housewife Unemployed Employed		219 (9.3) 1151 (49.0) 252 (10.7) 726 (31.0)	
Body weight (kg) at booking	2,546		69.6 ± 14.6
Height (m)	2,546		1.6 ± 0.1
BMI (kg/m^2^) at booking	2,546		27.7 ± 5.6
Previous history of GDM	2,047	422 (20.6)	
Family history of type 2 diabetes	2,546	754 (29.6)	
**Oral Glucose Tolerance Test (OGTT) results in mmol/L** (at 24 to 32 weeks of gestation)			
Fasting plasma glucose (FPG)	1,188		4.6 ± 0.4
1-h OGTT	1,548		8.0 ± 1.9
2-h OGTT	2,443		6.5 ± 1.6

BMI, body mass index; kg, kilograms; m, meters; mmol/L, millimoles per liter.

a Total number of participants who had data for a particular variable.

### GDM incidence by different diagnostic criteria

**Figure 1:**
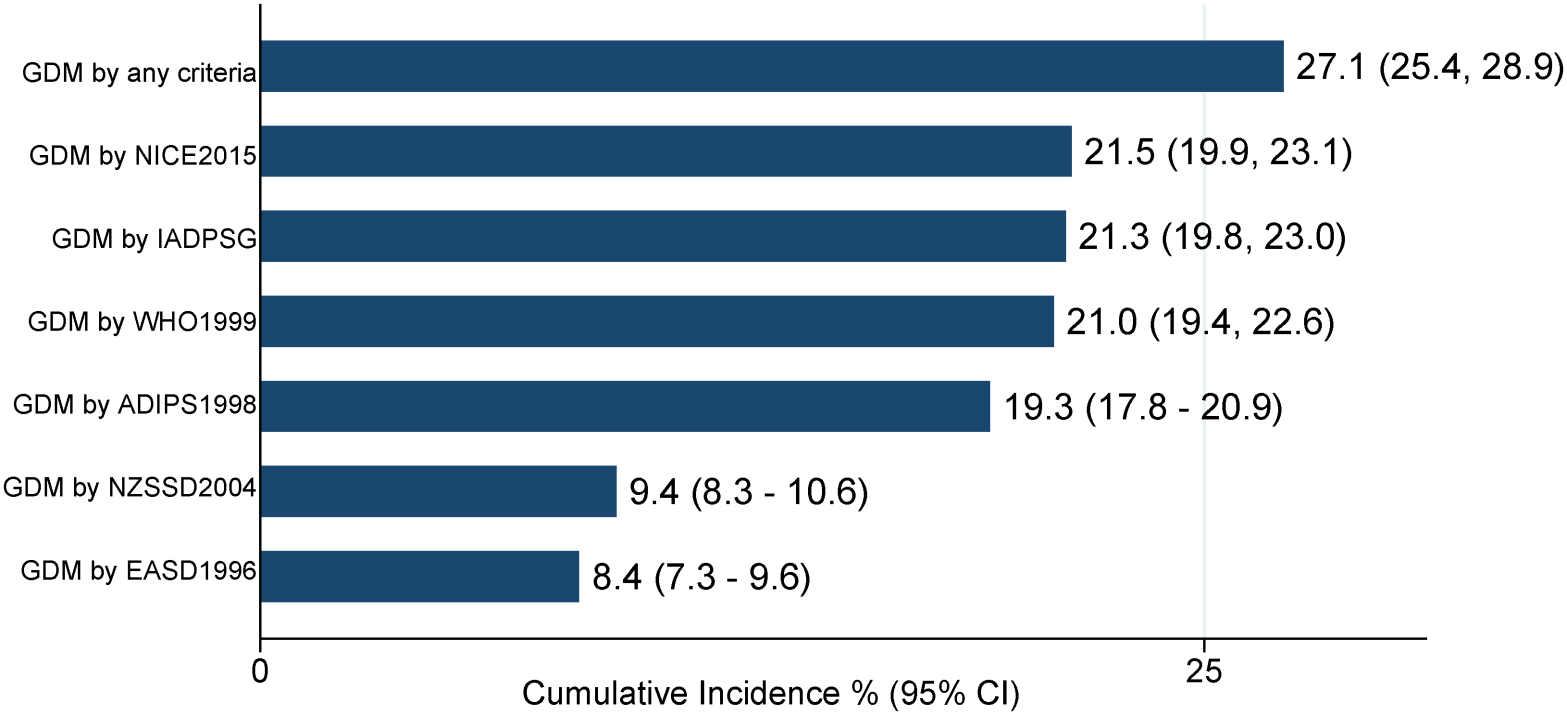
GDM cumulative incidence among pregnant women using six GDM diagnostic criteria (N = 2,546). IADPSG, International Association of Diabetes and Pregnancy Study Groups; NICE, National Institute for Health and Clinical Excellence; WHO, World Health Organization; ADIPS, Australasian Diabetes in Pregnancy Society; EASD, European Association for the Study of Diabetes; NZSSD, New Zealand Society for the Study of Diabetes.

**Figure 1** compares the GDM cumulative incidence diagnosed by the six different criteria. The NICE 2015 and the IADPSG criteria were the most inclusive in this population, showing GDM incidence rates of 21.5% (95% CI: 19.9, 23.1) and 21.3% (95% CI: 19.8, 23.0), respectively. The EASD 1996 criteria showed the lowest GDM incidence rate of 8.4% (95% CI: 7.3, 9.6).

### GDM risk factors by different criteria

**Table 3** shows the distribution of GDM risk factors according to the six diagnostic criteria. Compared with the non-GDM group identified by each diagnostic criterion, most of the risk factors were significantly higher among those with GDM (p < 0.005). An exception is seen for family history of type 2 DM using the EASD 1996 criteria (p > 0.05). **Table 4** also shows the distribution of the GDM risk factors according to the six diagnostic criteria, but, in this instance, the comparison group was non-GDM participants identified by all six criteria. Here, also, risk factors were significantly higher among those with GDM (p < 0.001) across all criteria.

**Table 3:** Risk factors distribution according to participants’ GDM status (diagnosed by six criteria) N = 2546.

	IADPSG		NICE 2015		WHO 1999		ADIPS 1998		EASD 1996		NZSSD 2004		Any criteria	
Variables^a^	GDM, n = 543 (21.3%)	NO GDM, n = 2003 (78.7%)	GDM, n = 547 (21.5%)	NO GDM, n = 1,999 (78.5%)	GDM, n = 535 (21.0%)	NO GDM, n = 2,011 (79.0%)	GDM, n = 492 (19.3%)	NO GDM, n = 2,054 (80.7%)	GDM, n = 215 (8.4%)	NO GDM, n = 2,331 (91.6%)	GDM, n = 240 (9.4%)	NO GDM, n = 2,306 (90.6%)	GDM, n = 690 (27.1%)	NO GDM, n = 1,856 (72.9%)
**Age, mean (SD)**	32.4 (5.8)	30.0 (6.0)*	32.4 (5.7)	30.0 (6.0)*	32.4 (5.7)	30.0 (6.0)*	32.5 (5.7)	30.0 (6.0)*	33.1 (5.9)	30.2 (6.0)*	33.0 (6.0)	30.2 (6.0)*	32.1 (5.7)	29.9 (6.0)*
**Gravidity, mean (SD)**	3.9 (2.3)	3.4 (2.1)*	3.9 (2.2)	3.4 (2.1)*	4.0 (2.2)	3.4 (2.1)*	3.9 (2.3)	3.4 (2.1)*	4.2 (2.3)	3.5 (2.1)*	4.2 (2.4)	3.5 (2.1)*	3.9 (2.3)	3.4 (2.1)*
**BMI, mean (SD) ^b^**	29.4 (5.6)	27.3 (5.5)*	29.0 (5.5)	27.4 (5.5)*	29.1 (5.5)	27.4 (5.5)*	29.0 (5.4)	27.4 (5.6)*	29.5 (5.4)	27.5 (5.6)*	29.5 (5.2)	27.5 (5.6)*	29.1 (5.7)	27.2 (5.5)*
**Previous GDM, n (%)**	203 (45.2)	219 (13.7)*	188 (41.3)	234 (14.7)*	183 (40.9)	239 (15.0)*	173 (42.1)	249 (15.2)*	86 (48.9)	336 (18.0)*	97 (49.0)	325 (17.6)*	238 (41.5)	184 (12.5)*
**FHx of DM, n (%) ^c^**	194 (35.7)	560 (28.0)*	201 (36.8)	553 (27.7)*	194 (36.3)	560 (27.9)*	184 (37.4)	570 (27.7)*	73 (34.0)	681 (29.2) **^¥^**	91 (37.9)	663 (28.8)*	251 (36.4)	503 (27.1)*

^a^Column percentages were reported for the categorical variables; see **Table 2** for missingness of variables. *P-value < 0.005 and **^¥^** P-value > 0.05. Chi-square test was used for categorical variables and t-test for continuous variables. ^b^ BMI, body mass index; ^c^ FHx, family history of type 2 diabetes mellitus. IADPSG, International Association of Diabetes and Pregnancy Study Groups; NICE, National Institute for Health and Clinical Excellence; WHO, World Health Organization; ADIPS, Australasian Diabetes in Pregnancy Society; EASD, European Association for the Study of Diabetes; NZSSD, New Zealand Society for the Study of Diabetes.

**Table 4:** Risk factors distribution according to the GDM status (comparison is with no GDM by all criteria).

Variables^a^	NO GDM (all criteria)^b^, n = 1856	IADPSG GDM, n = 543 (22.6%)	NICE 2015 GDM, n = 547 (22.8%)	WHO 1999 GDM, n = 535 (22.4%)	ADIPS 1998 GDM, n = 492 (21.0%)	EASD 1996 GDM, n = 215 (10.4%)	NZSSD 2004 GDM, n = 240 (11.4%)
**Age, Mean (SD)**	29.9 (6.0)	32.4 (5.8)*	32.4 (5.7)*	32.4 (5.7)*	32.5 (5.7)*	33.1 (5.9)*	33.0 (6.0)*
**Gravidity, Mean (SD)**	3.4 (2.1)	3.9 (2.3)*	3.9 (2.2)*	4.0 (2.2)*	3.9 (2.3)*	4.2 (2.3)*	4.2 (2.4)*
**BMI, Mean (SD)**	27.2 (5.5)	29.4 (5.6)*	29.0 (5.5)*	29.1 (5.5)*	29.0 (5.4)*	29.5 (5.4)*	29.5 (5.2)*
**Previous GDM, n (%)**	184 (12.5)	203 (45.2)*	188 (41.3)*	183 (40.9)*	173 (42.1)*	86 (48.9)*	97 (49.0)*
**FHx of DM, n (%)**	503 (27.1)	194 (35.7)*	201 (36.8)*	194 (36.3)*	184 (37.4)*	73 (34.0)*	91 (37.9)*

^a^Column percentages were reported for the categorical variables; see **Table 2** for missingness of variables. ^b^ Women who were GDM negative using all the six criteria. *P-value < 0.001, P-value specified at 0.05 and shows comparison of a risk factor between GDM diagnosed by specified criteria and no GDM by all criteria. Chi-square test was used for categorical variables and t-test for continuous variables. IADPSG, International Association of Diabetes and Pregnancy Study Groups; NICE, National Institute for Health and Clinical Excellence; WHO, World Health Organization; ADIPS, Australasian Diabetes in Pregnancy Society; EASD, European Association for the Study of Diabetes; NZSSD, New Zealand Society for the Study of Diabetes.

### Agreement among the different GDM diagnostic criteria

**Table 5** compares the agreement between the diagnostic criteria in pairs. Agreement between the two most inclusive criteria (NICE 2015 and IADPSG criteria) was moderate, with Cohen’s kappa coefficient (k) of 0.66; p < 0.001. The highest agreement was between the NICE 2015 and WHO 1999 criteria (0.99; p < 0.001), whereas the lowest was between the NZSSD 2004 and WHO 1999 criteria (0.49; p < 0.001). The locally recommended IADPSG/WHO 2013 criteria had weak to moderate agreement with the other criteria, with Cohen’s kappa coefficient ranging from (k = 0.51; p < 0.001) to (k = 0.71; p < 0.001).

**Table 5:** Comparing agreement between diagnostic criteria (in pairs) using k statistics.

	IADPSG	NICE2015	WHO 1999	ADIPS 1998	EASD 1996	NZSSD 2004
IADPSG	**1.0**					
NICE 2015	0.66	**1.0**				
WHO 1999	0.64	0.99	**1.0**			
ADIPS 1998	0.71	0.91	0.89	**1.0**		
EASD 1996	0.51	0.50	0.50	0.56	**1.0**	
NZSSD 2004	0.55	0.52	0.49	0.61	0.94	**1.0**

Cohen’s kappa coefficient (k) interpretation for agreement (35); 0–0.20, none; 0.21–0.39, minimal; 0.40–0.59, weak; 0.60–0.79, moderate; 0.80–9.0, strong; >9.0, almost perfect/perfect. P-values were <0.001 for all the comparisons (k statistics). IADPSG, International Association of Diabetes and Pregnancy Study Groups; NICE, National Institute for Health and Clinical Excellence; WHO, World Health Organization; ADIPS, Australasian Diabetes in Pregnancy Society; EASD, European Association for the Study of Diabetes; NZSSD, New Zealand Society for the Study of Diabetes.

1.0 – constant.

### Sensitivity analysis

**Table 6** stratifies the GDM cumulative incidence by the number of non-missing OGTT readings used for diagnosis, i.e., those having at least one versus those having at least two OGTT readings. GDM incidence rate ranged from 8.4% using the EASD 1996 criteria to 21.5% using the NICE 2015 criteria among those who had at least one reading and 8.8% using the EASD 1996 criteria to 22.3% using the NICE 2015 criteria among those who had at least two readings. There was minimal change in the GDM incidence between these two groups. The NICE criteria remained the most inclusive in both groups; however, the WHO 1999 criteria were slightly more inclusive than the IADPSG criteria in the group having at least two non-missing readings [21.9 (20.2, 23.5) vs. 21.6 (20.0, 23.3), respectively].

**Table 6:** GDM criteria-specific cumulative incidence stratified by the number of OGTT readings used for diagnosis.

Non-missing OGTT readings	IADPSG, n (%)	NICE 2015, n (%)	WHO 1999, n (%)	ADIPS 1998, n (%)	EASD 1996, n (%)	NZSSD 2004, n (%)	Any criteria, n (%)
**Having at least 1 reading (N = 2,546)**	21.3 (19.8, 23.0)	21.5 (19.9, 23.1)	21.0 (19.4, 23.0)	19.3 (17.8, 20.9)	8.4 (7.3, 9.6)	9.4 (8.3, 10.6)	27.1 (25.4, 28.9)
**Having at least 2 readings (N = 2,449)**	21.6 (20.0, 23.3)	22.3 (20.6, 24.0)	21.9 (20.2, 23.5)	19.9 (18.4, 21.6)	8.8 (7.7, 10.0)	9.6 (8.5, 10.9)	27.6 (25.9, 29.5)

IADPSG, International Association of Diabetes and Pregnancy Study Groups; NICE, National Institute for Health and Clinical Excellence; WHO, World Health Organization; ADIPS, Australasian Diabetes in Pregnancy Society; EASD, European Association for the Study of Diabetes; NZSSD, New Zealand Society for the Study of Diabetes.

## Discussion

This study showed that GDM incidence differed among the Emirati population in the UAE, ranging from 8.4% according to the EASD 1996 criteria to 21.5% according to the NICE 2015 criteria. The most inclusive GDM diagnostic criteria in our study population were the NICE 2015 and IADPSG criteria (WHO 2013), whereas the EASD 1996 and NZSSD 2004 criteria were the least inclusive in this population. The study also showed GDM risk factor distribution across all criteria, with most of them being significantly higher among patients diagnosed with GDM. Agreement among the six criteria using the Cohen’s kappa coefficient ranged from weak to almost perfect (**Table 5**).

The GDM guidelines developed by the health authorities in the UAE mainly recommend using the IADPSG/WHO 2013 criteria (36, 37). However, there was evidence that different hospitals and doctors use different criteria for GDM diagnosis in the country (34). Although recommended, the IADPSG criteria were not the most inclusive in this study. This is contrary to the previous UAE studies (3, 29), although the NICE 2015 criteria were not developed at the time that they were conducted. This is the first study in the country to assess the newer NICE criteria. Our GDM incidence by the IADPSG criteria was comparable to that found in a study in Qatar (21.5%). However, the Qatar study showed that the NICE criteria were less inclusive than the IADPSG criteria (27). The higher inclusivity of the NICE criteria was an unexpected and interesting finding in our study. The IADPSG was, however, more inclusive than the remaining four criteria.

Similar to our study, many studies (26, 28, 38–40) have found that the IADPSG criteria (new WHO 2013) identify more GDM cases than the former WHO 1999 criteria, although the increase in our study is by eight GDM cases only (**Supplementary S1**), and, following sensitivity analysis, the WHO 1999 was slightly more inclusive among those having at least two OGTT readings. In contrast, only a few studies (24, 41, 42) showed that the former WHO criteria diagnose more GDM cases. The IADPSG also identified more GDM cases than the Australian (ADIPS 1998), European (EASD 1996), and New Zealand (NZSSD 2004) criteria. This finding was supported by several studies, as shown in the meta-analysis conducted by Saeedi et al. (28).

Unlike in some settings (43–45), our study had shown a general reduction in the GDM incidence in the UAE using different criteria. The IADPSG criteria showed a GDM incidence of 21.3%, which was lower than the previous 37.7% (4) and 45.3% (29) shown in other studies in the country using the same criteria in previous years. Still, our GDM incidence was much higher than the regional average (13.0%) (46). GDM incidence rates were also reduced using the other diagnostic criteria compared with previous studies. The estimated criteria-specific GDM incidence applying the WHO 1999, ADIPS 1998, EASD 1996, and NZSSD 2004 was lower than that found in previous studies (4, 29) in a similar population when compared with GDM incidence by corresponding criteria. This reduction could be due to some factors and may not necessarily reflect actual GDM incidence reduction in the general population. It is important to note that the previous studies were conducted among multi-ethnic groups. In addition, in this study, we included those with at least one OGTT reading, and this could have underestimated the incidence in general. Moreover, the NICE 2015 has not been assessed in this population before.

This study also showed GDM risk factor distribution among participants. Patients with GDM (as diagnosed by any criteria) were found to be significantly older, more gravid, have higher BMI, have more history of GDM, and have a family history of type 2 DM. This is supported by studies regionally (27) and globally (47). An exception was seen in the family history of type 2 DM in the EASD 1996 criteria (p > 0.05). However, this was also significant when compared with non-GDM group diagnosed by all criteria. In general, EASD 1996 and NZSSD 2004, the two least inclusive criteria, identified more participants with higher GDM risk factors. This is likely because they employ more strict criteria (higher cutoff for 2-h OGTT), hence identifying higher-risk patients.

Assessing the criteria compatibility, the IADPSG and NICE 2015 criteria together identified 400 (15.7%) patients with GDM in our population. This is in close agreement with the 14% identified in another regional study using the same two criteria (27). Furthermore, in our study, the NICE 2015 criteria diagnose more cases with the former WHO 1999 and ADIPS 1998 criteria than with the IADPSG criteria (**Supplementary S1**). This might be partially attributed to the fact that, among the six criteria that we assessed, only the IADPSG criteria utilizes the 1-h OGTT for diagnosis.

Cohen’s kappa coefficient showed that the criteria with the highest agreement were the NICE 2015 and WHO 1999 criteria, and the lowest were the NZSSD 2004 and WHO 1999 criteria. The NICE 2015 and IADPSG (the two most inclusive criteria) had only moderate agreement (0.66). This is like the study in Qatar where these two criteria had a kappa coefficient of 0.67 (27). The IADPSG, the locally recommended criteria, had weak to moderate agreement with the other criteria. This has mostly been the case in previous studies in the country (3, 29). These discrepancies were concerning, especially if different doctors and hospitals in the country use different GDM diagnostic criteria. GDM incidence across the country could not be combined accurately. We recommend further studies to assess the criteria commonly used by doctors in the country.

GDM incidence as diagnosed by any of the six criteria collectively was 27.1%. Following comparison of this with each criterion separately, the percentage of GDM cases missed by each of the six criteria was noted (**Supplementary S2**). The two most inclusive criteria, the NICE 2015 and IADPSG criteria, missed 20.7% and 21.3% of GDM cases diagnosed by the other criteria combined, whereas the two least inclusive, the NZSSD 2004 and EASD 1996 criteria, missed up to 65.2% and 68.8% GDM cases, respectively. Studies have shown that missing GDM cases could lead to increase burden of adverse perinatal outcomes (24, 48, 49). Although increase workload and cost of management have been associated with using more inclusive GDM diagnostic criteria (50, 51), on the other hand, the health and economic burden of having missed GDM cases is substantial (52–54). This makes the unification of GDM diagnostic criteria a priority using the most suitable for each population.

Following the study results, we recommend the unification of GDM diagnostic criteria in the UAE population. We advise withdrawing the use of the least inclusive criteria. The NICE 2015 is currently a strong contender to the locally recommended IADPSG criteria for diagnosing GDM. It has already been adopted by some doctors probably due to its simpler protocol (no 1-h OGTT used) is its cost-effectiveness as shown in some studies (24, 55). On the other hand, the IADPSG criteria is the only GDM criteria that were developed on the basis of the risk of adverse perinatal outcomes (56). Our study had shown that these two criteria were on par with each other in terms of inclusivity in GDM diagnosis among the study population and they do not have strong agreement with each other; hence, we recommend further studies to assess which criteria is most suitable for this population based on its risk of adverse outcomes.

The main strength of our study was the large representative population that increased the study power and generalizability of the findings and minimized estimate errors (57). The Mutaba’ah study is the largest prospective mother and child cohort study in the UAE, which provides data on maternal and child health from conception to adolescence. Our main limitation was that we did not have all three OGTT readings for all the participants, which might lead to the underestimation of incidences. In addition, those with all three could not be analyzed separately as the analysis would not be adequately powered. However, sensitivity analysis was performed among those with at least one and those with at least two readings providing for a more robust result. Prevention of adverse perinatal outcomes by different GDM diagnostic criteria was also not evaluated in this study.

## Conclusions

Our findings showed discrepancies among the GDM diagnostic criteria in the UAE Emirati population, with GDM incidence ranging from 8.4% to 21.5% as diagnosed by the six assessed criteria. The NICE 2015 criteria, followed by the IADPSG/WHO 2013 criteria, were the most inclusive criteria. These two criteria had a moderate agreement (Cohen’s kappa coefficient of 0.66). The locally recommended IADPSG criteria had weak to moderate agreements with the other five criteria.

This study has highlighted the need to unify GDM diagnostic criteria in this population, especially because the agreement with the recommended criteria is not optimal. Following our results, we recommend reviewing the use of IADPSG versus the NICE 2015 GDM criteria in this population. Further research is needed to assess doctors’ current practice. Moreover, longitudinal data on maternal and neonatal outcomes collected within the Mutaba’ah study will explore the optimal GDM criteria based on the risk of adverse perinatal outcomes in this population.

## Data availability statement

The data presented in this study can be made available on request from The Mutaba’ah Study. Approval from the research ethics committee may be required.

## Ethics statement

The study was reviewed and approved by the Abu Dhabi Health Research and Technology Ethics Committee (DOH/CVDC/2022/72). The participants provided their written informed consent to participate in this study.

## Author contributions

Conceptualization: MB, LA, RA-R, and FA-M. Methodology: MB, LA, IE, TL, JA, RA-R, and FA-M. Formal analysis: MB, LA, and FA-M. Investigation: MB, LA, IE, TL, RA-R, JA, and FA-M. Resources: LA, TL, JA, and FA-M. Data curation: MB and LA. Original manuscript draft preparation: MB. Manuscript review and editing: MB, LA, IE, TL, RA-R, JA, and FA-M. Visualization: MB, LA, IE, TL, RA-R, JA, and FA-M. Supervision: LA, IE, RA-R, and FA-M. Project administration: MB, LA, IE, TL, RA-R, JA, and FA-M. All authors have read and agreed to the published version of the manuscript.

## Supplementary material

Supplementary S1 Tables showing participants as classified by a pair of criteria.

Supplementary S2 Tables showing missed GDM cases by specific criteria when compared with other criteria.
