# Supplementary material for: Incidence of gestational diabetes mellitus in the United Arab Emirates; comparison of six diagnostic criteria: The Mutaba’ah Study
Source: Front Endocrinol (Lausanne). 2022 Dec 12;13:1069477. doi: 10.3389/fendo.2022.1069477 (PMC9791114; doi:10.3389/fendo.2022.1069477)
Supplement: Supplementary S1 — Tables showing participants as classified by a pair of criteria. [file DataSheet_1.pdf]

Supplementary Material

Tables showing participants classified by a pair of criteria (IADPSG & NICE combinations)

Tabulation of GDM_by_IADPSG GDM_by_NICE2015

GDM patients diagnosed by IADPSG	GDM patients diagnosed by NICE2015		
	No GDM	GDM	Total
No GDM	1856	147	2003
GDM	143	400	543
Total	1999	547	2546

Tabulation of GDM_by_IADPSG GDM_by_WHO1999

GDM patients diagnosed by IADPSG	GDM patients diagnosed by WHO1999		
	No GDM	GDM	Total
No GDM	1856	147	2003
GDM	155	388	543
Total	2011	535	2546

Tabulation of GDM_by_IADPSG GDM_by_ADIPS1998

GDM patients diagnosed by IADPSG	GDM patients diagnosed by ADIPS1998		
	No GDM	GDM	Total
No GDM	1908	95	2003
GDM	146	397	543
Total	2054	492	2546

Tabulation of GDM_by_IADPSG GDM_by_EASD1996

GDM patients diagnosed by IADPSG	GDM patients diagnosed by EASD1996		
	No GDM	GDM	Total
No GDM	2003	0	2003
GDM	328	215	543
Total	2331	215	2546

Tabulation of GDM_by_IADPSG GDM_by_NZSSD2004

GDM patients diagnosed by IADPSG	GDM patients diagnosed by NZSSD2004		
	No GDM	GDM	Total
No GDM	2003	0	2003
GDM	303	240	543
Total	2306	240	2546

Tabulation of GDM_by_NICE2015 GDM_by_WHO1999

GDM patients diagnosed by NICE2015	GDM patients diagnosed by WHO1999		
	No GDM	GDM	Total
No GDM	1999	0	1999
GDM	12	535	547
Total	2011	535	2546

Tabulation of GDM_by_NICE2015 GDM_by_ADIPS1998

GDM patients diagnosed by NICE2015	GDM patients diagnosed by ADIPS1998		
	No GDM	GDM	Total
No GDM	1988	11	1999
GDM	66	481	547
Total	2054	492	2546

Tabulation of GDM_by_NICE2015 GDM_by_EASD1996

GDM patients diagnosed by NICE2015	GDM patients diagnosed by EASD1996		
	No GDM	GDM	Total
No GDM	1999	0	1999
GDM	332	215	547
Total	2331	215	2546

Tabulation of GDM_by_NICE2015 GDM_by_NZSSD2004

GDM patients diagnosed by NICE2015	GDM patients diagnosed by NZSSD2004		
	No GDM	GDM	Total
No GDM	1988	11	1999
GDM	318	229	547
Total	2306	240	2546
