# Supplementary material for: Incidence of gestational diabetes mellitus in the United Arab Emirates; comparison of six diagnostic criteria: The Mutaba’ah Study
Source: Front Endocrinol (Lausanne). 2022 Dec 12;13:1069477. doi: 10.3389/fendo.2022.1069477 (PMC9791114; doi:10.3389/fendo.2022.1069477)
Supplement: Supplementary S2 — Tables showing missed GDM cases by specific criteria when compared with other criteria. [file DataSheet_2.pdf]

Supplementary Material

Tables showing missed GDM cases by specific criteria when compared to other criteria

Tabulation of GDM_by_Anycriteria & GDM_by_IADPSG

	GDM patients diagnosed by IADPSG		
GDM by Anycriteria	No GDM	GDM	Total
No GDM	1856	0	1856
	100.00	0.00	100.00
GDM	147	543	690
	21.30	78.70	100.00
Total	2003	543	2546
	78.67	21.33	100.00

First row has *frequencies* and second row has *row percentages*

Tabulation of GDM_by_Anycriteria & GDM_by_NICE2015

	GDM patients diagnosed by NICE2015		
GDM by Anycriteria	No GDM	GDM	Total
No GDM	1856	0	1856
	100.00	0.00	100.00
GDM	143	547	690
	20.72	79.28	100.00
Total	1999	547	2546
	78.52	21.48	100.00

First row has *frequencies* and second row has *row percentages*

Tabulation of GDM_by_Anycriteria & GDM_by_WHO1999

	GDM patients diagnosed by WHO1999		
GDM by Anycriteria	No GDM	GDM	Total
No GDM	1856	0	1856
	100.00	0.00	100.00
GDM	155	535	690
	22.46	77.54	100.00
Total	2011	535	2546
	78.99	21.01	100.00

First row has *frequencies* and second row has *row percentages*

Tabulation of GDM_by_Anycriteria & GDM_by_ADIPS1998

	GDM patients diagnosed by ADIPS1998		
GDM by Anycriteria	No GDM	GDM	Total
No GDM	1856	0	1856
	100.00	0.00	100.00
GDM	198	492	690
	28.70	71.30	100.00
Total	2054	492	2546
	80.68	19.32	100.00

First row has *frequencies* and second row has *row percentages*

Tabulation of GDM_by_Anycriteria & GDM_by_EASD1996

	GDM patients diagnosed by EASD1996		
GDM by Anycriteria	No GDM	GDM	Total
No GDM	1856	0	1856
	100.00	0.00	100.00
GDM	475	215	690
	68.84	31.16	100.00
Total	2331	215	2546
	91.56	8.44	100.00

First row has *frequencies* and second row has *row percentages*

Tabulation of GDM_by_Anycriteria & GDM_by_NZSSD2004

	GDM patients diagnosed by NZSSD2004		
GDM by Anycriteria	No GDM	GDM	Total
No GDM	1856	0	1856
	100.00	0.00	100.00
GDM	450	240	690
	65.22	34.78	100.00
Total	2306	240	2546
	90.57	9.43	100.00

First row has *frequencies* and second row has *row percentages*
